# A Midgut Digestive Phospholipase A_2_ in Larval Mosquitoes,* Aedes albopictus *and* Culex quinquefasciatus*

**DOI:** 10.1155/2018/9703413

**Published:** 2018-05-15

**Authors:** Nor Aliza Abdul Rahim, Marlini Othman, Muna Sabri, David W. Stanley

**Affiliations:** ^1^Department of Paraclinical Sciences, Faculty of Medicine and Health Sciences, Universiti Malaysia Sarawak, 94300 Kota Samarahan, Sarawak, Malaysia; ^2^Department of Basic Medical Sciences, Faculty of Medicine and Health Sciences, Universiti Malaysia Sarawak, 94300 Kota Samarahan, Sarawak, Malaysia; ^3^United States Department of Agriculture, Agricultural Research Service, Biological Control of Insects Research Laboratory, 1503 S. Providence Road, Columbia, MO 65203, USA

## Abstract

Phospholipase A_2_ (PLA_2_) is a secretory digestive enzyme that hydrolyzes ester bond at* sn-2 *position of dietary phospholipids, creating free fatty acid and lysophospholipid. The free fatty acids (arachidonic acid) are absorbed into midgut cells.* Aedes albopictus *and* Culex quinquefasciatus *digestive PLA_2_ was characterized using a microplate PLA_2_ assay. The enzyme showed substantial activities at 6 and 8 *μ*g/*μ*l of protein concentration with optimal activity at 20 and 25 *μ*g/*μ*l of substrate concentration in* Aedes albopictus* and* Culex quinquefasciatus*, respectively. PLA_2_ activity from both mosquitoes increased in a linear function up to 1 hour of the reaction time. Both enzymes were sensitive to pH and temperature. PLA_2_ showed higher enzyme activities in pH 8.0 and pH 9.0 from* Aedes albopictus* and* Culex quinquefasciatus*, respectively, at 40°C of incubation. The PLA_2_ activity decreased in the presence of 5 mM* (Aedes albopictus)* and 0.5 mM* (Culex quinquefasciatus)* site specific PLA_2 _inhibitor, oleyloxyethylphosphorylcholine. Based on the migration pattern of the partially purified PLA_2_ on SDS-PAGE, the protein mass of PLA_2_ is approximately 20–25 kDa for both mosquitoes. The information on PLA_2_ properties derived from this study may facilitate in devising mosquitoes control strategies especially in the development of inhibitors targeting the enzyme active site.

## 1. Introduction

Phospholipase A_2_ (PLA_2_) hydrolyzes the* sn-2* ester bond in phospholipids (PLs) [1]. These enzymes make up a large superfamily of proteins that act in a very wide variety of physiological and pathophysiological actions. PLA_2_ actions include digestion of dietary lipids, remodelling cellular membranes, host immune defenses, signal transduction via production of various lipid mediators, and, in the case of platelet activating factor, inactivation of a lipid mediator. Research into noncatalytic PLA_2_s and into PLA_2_ receptors and binding proteins reveals entirely new biological actions in which PLA_2_ acts as a ligand rather than a catalytic enzyme [2, 3]. Here, we focus attention on PLA_2_ associated with digestion.

Lipid digestion and absorption take place in the insect midguts. Midgut cells produce and secrete lipases that digest dietary neutral lipids, such as triacylglycerols. PLA_2_s are responsible for two separate actions in insect physiology. For one, PLA_2_s hydrolyze a fatty acid from the* sn*-2 position of dietary PLs. Typically, the fatty acids esterified to the* sn*-2 positions are C18 and C20 PUFAs. These fatty acids include linoleic acid, 18:2n-6, and linolenic acid, 18:3n-3, one or the other of which is strictly essential nutritional requirements for most insects and nearly all vertebrates. Hence, midgut PLA_2_s are necessary for insects to meet one of their essential nutritional needs. A few insect and invertebrate species express a Δ^−12^ desaturase that inserts a double bond into oleic acid (18:1n-9), yielding 18:2n-6 and obviating the nutritional requirement [4–6]. The desaturation and elongation pathways necessary to convert C18 PUFAs to their C20 counterparts have been documented in several insect species [7, 8], from which we infer insects are able to meet all fatty acid requirements via dietary linoleic and linolenic acids, coupled to the desaturase/elongation pathways. The second important PLA_2_ action contributes to digestion of dietary neutral lipids. Vertebrates, but not insects, produce bile salts that facilitate lipid digestion by solubilizing neutral lipids. In each PLA_2_ reaction, hydrolysis of the* sn-2 *fatty acid from PL leads to a free fatty acid and to a lysoPLs and these lipids act as the necessary solubilizers that aid lipase digestion of neutral lipids.

PLA_2_ is the key enzyme responsible in the hydrolysis of arachidonic acid which acts as precursors to lipid mediators such as prostaglandins [9]. PLA_2_s occur abundantly in venoms [10], in pancreatic juices of mammals, and in synovial fluids [11]. While PLA_2_s are well characterized in terms of protein and gene structures in mammalian physiology, there is relatively little information on the characteristics of insect digestive PLA_2_. Nonetheless, these enzymes are very important in insect biology and they may become functional targets in some pest management programs. Seen in this light, there is a real need for new knowledge on insect digestive PLA_2_s. In this paper, we begin to address that need. Here, we report on the presence and the characteristics of a midgut PLA_2_ in larvae of the mosquitoes,* Aedes albopictus* and* Culex quinquefasciatus*.

## 2. Materials and Methods

### 2.1. Insect

The larvae of* Aedes albopictus *and* Culex quinquefasciatus *were collected from Kampung Semerah Padi, Petra Jaya, Kuching (1°34′59.3^″^N, 110°19′48.2^″^E). The larvae were collected with ovitraps filled with a cow-grass infusion solution following methods described by Tang et al. [12].

Ten ovitraps were placed near housing areas at Kampung Semerah Padi, Petra Jaya. The ovitraps were collected and replaced with new ones every week. The larvae collected were pooled together and fed with fish food until the 4th instar. The larvae were identified as* Aedes albopictus *and* Culex quinquefasciatus *[13] and midguts were isolated. A larva to be dissected was placed on a glass plate and the water surrounding the larvae was blotted to dry. The midgut was removed by using a pair of forceps. By holding the thorax with forceps, the 8th abdominal segment was gently pulled using the other forceps so that the entire alimentary canal was drown out. The anal papillae, siphon, and Malphigian tubule attaching to the midgut were removed by pinching with the forceps.

### 2.2. PLA_2_ Source Preparation

Midgut samples were homogenized in 200 *μ*l buffer (0.1 M Tris[hydroxymethyl]aminoethane, pH 8; Sigma) mixed with 2 mM phenylthiourea (PTU, Sigma) by using a Bio Masher (Optima, Inc., USA). The homogenates were centrifuged at 735*g* for 3 minutes, then at 11,750*g* for 10 minutes. The supernatants were collected and used as the enzyme preparation.

### 2.3. Phospholipase A_2_ Assay

PLA_2_ substrate, 4-nitro-3-(octanoyloxy)benzoic acid (NOB; Enzo Life Sciences, Switzerland), was prepared following Nenad et al. [14] and Beghini et al. [15] with several modifications. The substrate was diluted with chloroform to 50 mg/ml. 20 *μ*l (1 mg) aliquots were distributed into Eppendorf tubes and all of the moisture was evaporated to dryness. The dry residue was stored at −20°C. Immediately before the assay, the substrate was resuspended in 1 ml of acetonitrile. The suspension was vortexed until all the substrate dissolved.

A standard PLA_2_ enzyme assay using 96-well plates was conducted following methods by Beghini et al. [15]. The standard assay mixture contains substrate (NOB), enzyme source, and buffer (0.1 M Tris buffer, pH 8) made up of a 200 *μ*l of mixture in each well. After the addition of the enzyme source, the microplate was incubated (Asys Thermostar) for 40 minutes at 40°C. The NOB, through hydrolysis of an ester bond, will convert into a chromophore (4-nitro-3-hydroxy-benzoic acid). The absorbance of chromophore concentration produced was quantified using a microplate reader at 405 nm. The effects of Ca^2+^, substrate and protein concentration, incubation time, pH, and temperature were investigated by varying each parameter.

### 2.4. Localizing the PLA_2_ in Larvae

The homogenates were prepared from three different sections of the individual larvae. Three groups, A, B, and C, consisted of gut-free larval bodies, guts and contents, and isolated gut contents. Alimentary canals were removed by pulling out the eighth abdominal segment of the larvae with forceps, while holding its thorax with second forceps. The individual gut was quickly removed and placed into an Eppendorf tube containing 0.1 M Tris buffer and 2 mM PTU (Group B). The remaining bodies were collected into different tubes containing the same buffer (Group A). Isolated gut contents were obtained by separating the gut contents (Group C) from forty individual guts. All samples were prepared for enzyme assay as described in Table 1.

### 2.5. The Influence of Ca^2+^ on PLA_2_ Activity

We conducted reactions in the presence of three different buffers: (1) Tris buffer with no additions; (2) Tris buffer amended with 5 mM CaCl_2_; (3) Tris buffer amended with the Ca^2+^ chelator 5 mM EGTA (ethylene-glycol-bis (*β*-aminoethyl ether)-N,N,N′,N′-tetraacetic acid). Each buffer was used during larvae dissection, homogenization, and enzyme activity assay.

### 2.6. Characterizing the Mosquito Digestive PLA_2_

All experiments used midguts plus contents as the enzyme preparations. The influence of substrate and protein concentration, incubation time, pH, temperature, and the effect of site specific inhibitor for PLA_2_ and OOPC on PLA_2_ activity were assessed by varying each of the parameters.

### 2.7. Gel Electrophoresis

Estimation of this digestive PLA_2_ from* Aedes albopictus* and* Culex quinquefasciatus* was performed according to the tricine-polyacrylamide gel electrophoresis (Tricine-SDS-PAGE) method by Schägger and von Jagow [16].

Migration of digestive PLA_2_ was compared to the standard protein markers (Sigma) in a range of 26.6 kDa to 1.06 kDa. The protein marker consisted of triosephosphate isomerase from rabbit muscle (26.6 kDa), myoglobin from horse heart (17.0 kDa), *α*-lactalbumin from bovine milk (14.2 kDa), aprotinin from bovine lung (6.5 kDa), insulin chain B, oxidized, bovine (3.496 kDa), and bradykinin (1.06 kDa). The protein bands on the electrophoresis gel were directly visualized by using silver staining according to the method by Gromova and Celis [17].

### 2.8. Statistical Analysis

Data were reported as means ± SEM of *n* experiments as appropriate. The significance of difference between groups was assessed using one-way analysis of variance (ANOVA) followed by Tukey's multiple comparison test to determine the significant group. The confidence limit for significance was *p* ≤ 0.05.

## 3. Results

### 3.1. Localizing the PLA_2_ Enzyme in Larvae

**Figure 1:**
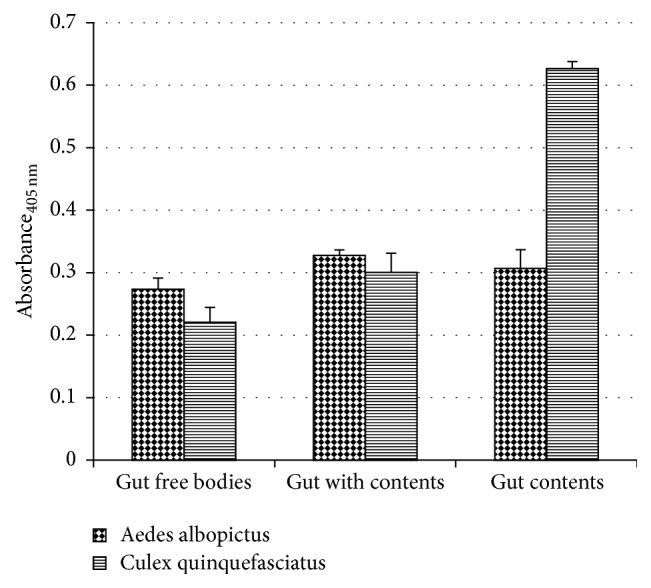
The PLA_2_ activity in different preparations of* Aedes albopictus* and* Culex quinquefasciatus *larvae. 10 *μ*g/*μ*l of protein concentration was reacted with 10 *μ*g/*μ*l of substrate concentration. Each histogram bar shows the mean ± SEM of triplicates from a single experiment representative of at least two experiments.

To determine the appropriate preparation for characterization of PLA_2_, three samples were prepared. Substantially high PLA_2_ enzyme activity from* Aedes albopictus *was recorded in gut plus content preparation (*M* = 0.328, SEM = 0.00) and gut content preparation (*M* = 0.3067, SEM = 0.01). There were no significant differences (*F*(2,6) = 5.161, *p* = 0.05) in PLA_2_ activity between all the preparations. In contrast, significantly higher PLA_2_ activity was observed in* Culex quinquefasciatus' *gut content preparation (*M* = 0.627, SEM = 0.01) (Figure 1). Similarly, lower PLA_2_ activity was recorded in gut-free bodies of* Aedes albopictus* (*M* = 0.2733, SEM = 0.01) and* Culex quinquefasciatus* (*M* = 0.220, SD = 0.02). The gut plus content was used as an enzyme source in all subsequent experiments.

### 3.2. Calcium Ion (Ca^2+^) Dependency

**Figure 2:**
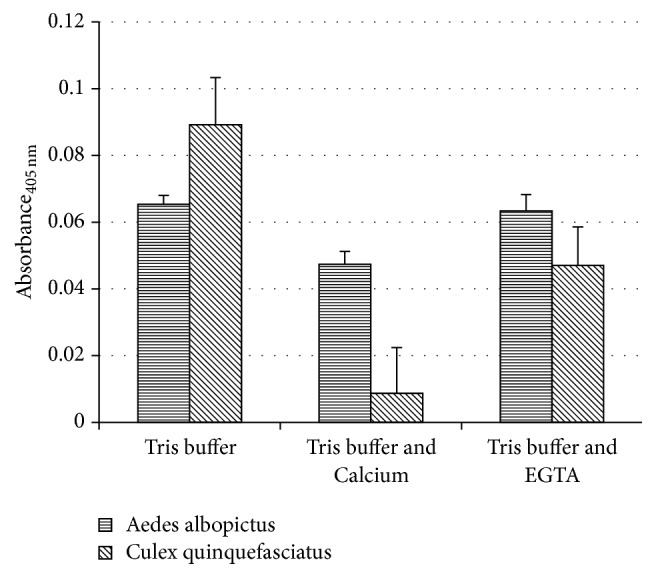
The PLA_2_ activity of* Aedes albopictus* and* Culex quinquefasciatus *in three different buffers. 10 *μ*g/*μ*l of protein concentration was reacted with 10 *μ*g/*μ*l of substrate concentration. Each histogram bar shows the mean ± SEM of triplicates from a single experiment representative of at least two experiments.

*Aedes albopictus *PLA_2_ activity was significantly higher in the buffer containing EGTA (*M* = 0.063, SEM = 0.01) and Tris buffer (*M* = 0.065, SEM = 0.00), while, for* Culex quinquefasciatus,* the PLA_2_ activity was significantly high in Tris buffer (*M* = 0.089, SEM = 0.01) compared to enzyme activity in Tris buffer with additional calcium (*M* = 0.0087, SEM = 0.01) (Figure 2). These results suggest that the mosquito larval preparation is independent of Ca^2+^. Subsequent experiments were conducted in Tris buffer with no added Ca^2+^.

### 3.3. Characterization of PLA_2_ Enzyme

**Figure 3:**
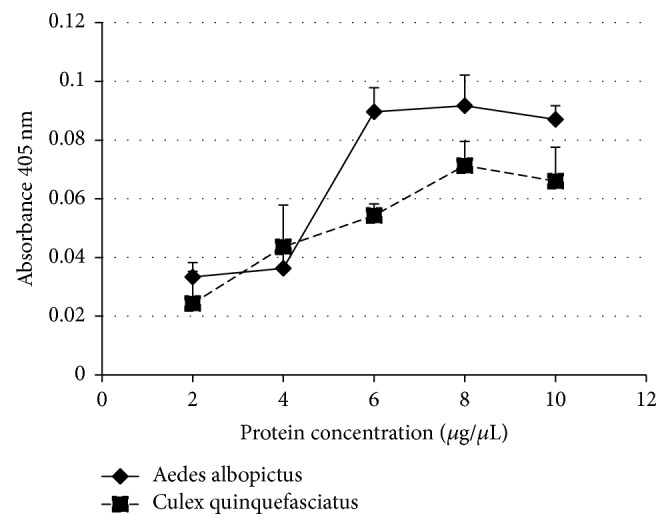
The influence of protein concentration on* Aedes albopictus *and* Culex quinquefasciatus *PLA_2_ activity. 10 *μ*g/*μ*l of substrate concentration was used to react with each of the protein concentrations, respectively. Each point represents the mean ± SEM of triplicates from a single experiment representative of at least two experiments.

The PLA_2_ activities from* Aedes albopictus* and* Culex quinquefasciatus* showed a similar trend where the optimum enzyme activities were recorded at 6–8 *μ*g/*μ*L of protein (Figure 3).* Aedes albopictus *PLA_2_ activity was fairly low at 2 and 4 *μ*g/*μ*l and then increased to a high level at 6 *μ*g/*μ*L (*M* = 0.090, SEM = 0.01) and 8 *μ*g/*μ*L (*M* = 0.091, SEM = 0.01) before it slightly declined at 10 *μ*g/*μ*L (*M* = 0.087, SEM = 0.00). Similarly, the* Culex quinquefasciatus *PLA_2_ activity increased from 2 *μ*g/*μ*L (*M* = 0.0243,   SEM = 0.01) of protein until it reached the highest activity at 8 *μ*g/*μ*L (*M* = 0.0713, SEM = 0.01). For subsequent experiment, 6 *μ*g/*μ*L of protein was used as an enzyme source for PLA_2_ assay in* Aedes albopictus *while 8 *μ*g/*μ*L was used as an enzyme source for PLA_2_ assay in* Culex quinquefasciatus*.

**Figure 4:**
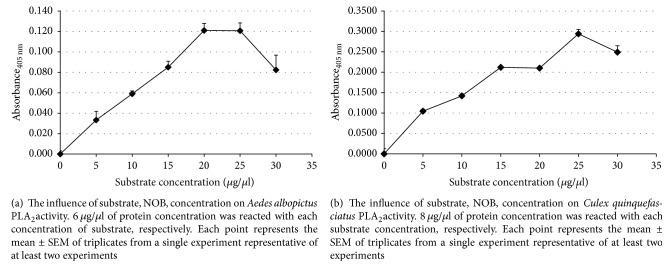


The PLA_2_ activities from mosquitoes,* Aedes albopictus *and* Culex quinquefasciatus*, were increased with increasing concentration of substrate until the enzyme concentration becomes a limiting factor in the reaction. The* Aedes albopictus* PLA_2_ activity increased in a linear manner with increasing substrate concentrations, up to 20 *μ*g/*μ*l (*M* = 0.121, SEM = 0.01) (Figure 4(a)).

PLA_2_ activity of* Culex quinquefasciatus* also increased from lower concentration, 5 *μ*g/*μ*L (*M* = 0.103, SEM = 0.00), until it reached its optimal activity at 25 *μ*g/*μ*L (*M* = 0.290, SEM = 0.01) (Figure 4(b)). There was no significant increase of enzyme activity at 30 *μ*g/*μ*L for both mosquitoes PLA_2_. Our standard concentration of substrate was 10 *μ*g/*μ*l for all subsequent experiments.

**Figure 5:**
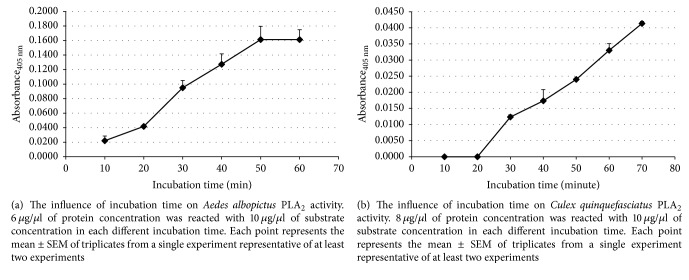


The PLA_2_ of* Aedes albopictus *was fairly low up to 20-minute incubations (*M* = 0.042, SEM = 0.01) and then increased substantially up to 50-minute incubation time (*M* = 0.161, SEM = 0.02). There was still another increase at 50 minutes (*M* = 0.161, SEM = 0.02) (Figure 5(a)). The enzyme activity remained constant at 60 minutes of incubation time.

On the other hand, there is no PLA_2_ activity of* Culex quinquefasciatus* recorded in the first 20 minutes of incubation time. Then, the enzyme activity continued to increase steadily even after an hour (*M* = 0.033, SD = 0.00) (Figure 5(b)). We used 40-minute incubations in all experiments.

**Figure 6:**
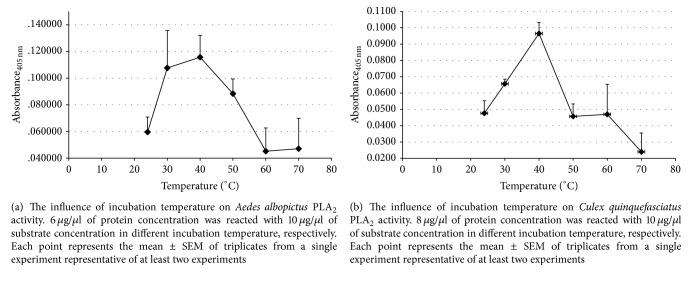


The digestive PLA_2_ was sensitive to temperature. The reaction mixtures were incubated in a range of 24°C (room temperature) to 70°C. The* Aedes albopictus* and* Culex quinquefasciatus *PLA_2_ activity increased in a linear way from room temperature to a peak at 40°C (*M* = 0.116, SEM = 0.01; *M* = 0.097, SEM = 0.01) (Figures 6(a) and 6(b)). At higher temperatures (60–70°C) the enzyme activity declined. Our standard incubation temperature was set at 40°C.

**Figure 7:**
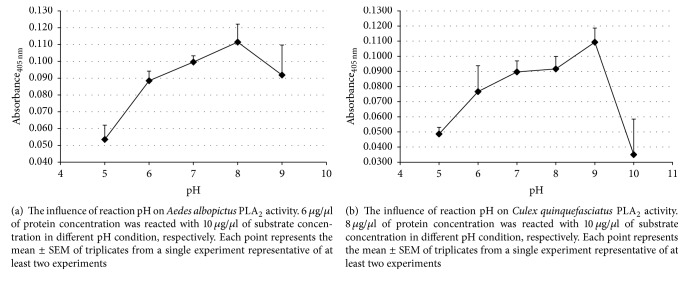


pH of the reaction mixtures influenced PLA_2_ (Figures 7(a) and 7(b)). The PLA_2_ activity from* Aedes albopictus* and* Culex quinquefasciatus* increased from acidic condition to a mild alkaline condition.* Aedes albopictus *PLA_2_ increased gradually from pH 5.0 (*M* = 0.067, SEM = 0.01) until it reached a maximum PLA_2_ activity at pH 8.0 (*M* = 0.130, SEM = 0.01). At pH 9.0, the enzyme activity slightly decreased, but it is not statistically significant. Similarly,* Culex quinquefasciatus *reached its highest PLA_2_ activity at pH 9.0 (*M* = 0.1093, SEM = 0.01) before it dropped drastically at pH 10.0 (*M* = 0.035, SEM = 0.02). Post hoc multiple comparison (Tukey) analysis showed there was no significant difference in the enzyme activity from pH 6.0 to 9.0. Therefore, pH 8 was selected as a reaction pH for all subsequent reactions.

### 3.4. The Influence of OOPC on PLA_2_ Activity

**Figure 8:**
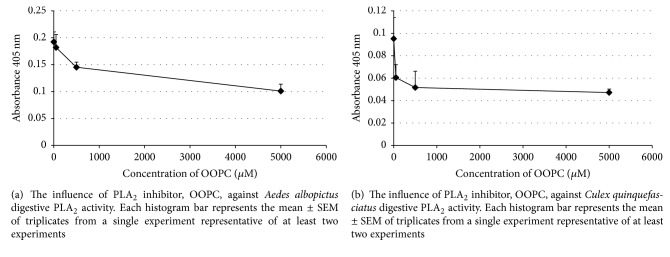


Reactions in the presence of 50 *μ*M to 500 *μ*M of OOPC led to a dose-related decline in PLA_2_ activity (Figures 8(a) and 8(b)). The PLA_2_ of* Aedes albopictus *was significantly inhibited in the presence of 5000 *μ*M of OOPC [*F*(3,8) = 5.886, *p* = 0.020]. However, the PLA_2_ of* Culex quinquefasciatus *was not statistically inhibited in the presence of PLA_2_ inhibitor, OOPC [*F*(2,6) = 3.651, *p* = 0.092].

### 3.5. Protein Mass Determination of Partially Purified Digestive PLA_2_

**Figure 9:**
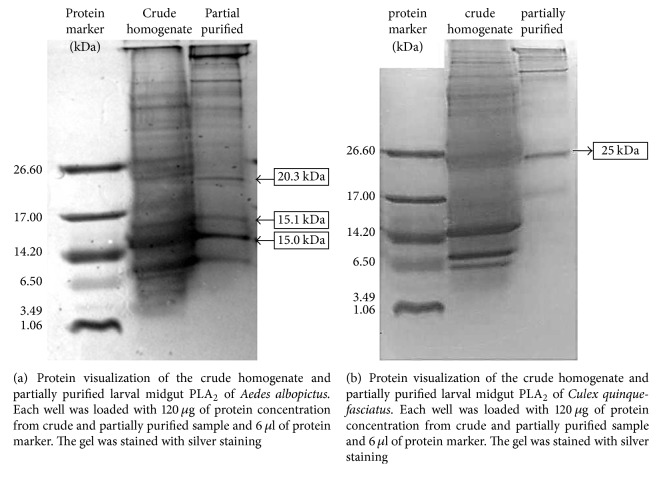


The protein electrophoretic profile of partially purified PLA_2_ from* Aedes albopictus* showed bands in different sizes which range from 14.6 to 20.3 kDa (Figure 9(a)) while for partially purified PLA_2_ from* Culex quinquefasciatus* it showed only one band which was at 25 kDa (Figure 9(b)). The presence of band at a similar size (25 kDa) suggests the presence of similar protein, which is the PLA_2_.

## 4. Discussion

In this paper, we report on the characterization of a digestive PLA_2_ in* Aedes albopictus *and* Culex quinquefasciatus *larvae. During our initial experiment, we compared the PLA_2_ activities in selected fractions of the alimentary canal. Higher PLA_2_ enzyme activity was recorded in the midgut plus content and isolated gut content preparations. Similar findings were reported for a related mosquito species,* Aedes aegypti*, where higher PLA_2_ enzyme activity was recorded in midgut plus content preparation. These findings suggested that midgut cells secrete more PLA_2_ than they store [18].

Secretory PLA_2_ is characterized as a low molecular weight molecule (13–55 kDa) [10, 19] that catalyzes substrate in full activity in the presence of calcium. sPLA_2_ differs from other PLA_2_ as it acts extracellularly [20]. The enzyme was secreted from the cells before catalyzing the substrate which usually occurs in the lumen of the insects' midgut [21].

The characterization of PLA_2_ in insects was assayed with radioactive substrate in the past. Here, we used a microplate assay using the chromogenic substrate, NOB. NOB is widely used in characterizing PLA_2_ from snake venom [22–24] and human serum [14]. Our PLA_2_ assays were performed on mosquito samples that have been partially enriched using Heparin column chromatography (1 ml HiTrap Heparin, Sigma, USA). This method successfully enriched the target PLA_2_ in primary screwworm preparations [25]. For this work, our simple microplate assay used only a small amount of protein and substrate to obtain an optimal PLA_2_ activity. This is an advantage for an investigation with limited amounts of protein sample. Since we collected our larval mosquito from the field, the number of individual larvae in each collection varied depending on their local environmental condition. The microplate assay is a practical but effective method to conduct our experiments with limited enzyme source.

Ca^2+^ is essential for both catalysis and binding of some enzymes to the substrate [26]. A study of Ca^2+^ requirement in primary screwworm PLA_2_ preparations showed that the enzyme activity was almost abolished in the presence of calcium chelator, EGTA. The PLA_2_ dependency on Ca^2+^ varied across species [21]. Previous studies on PLA_2_ from primary screwworm,* C. hominivorax *[25], robber flies,* Asilis *sp. [27], and adult tiger beetles,* Cicindela circumpicta *[28], showed strict Ca^2+^ requirement for catalysis. Several studies on PLA_2_ from venom sources such as rattlesnake,* Crotalus durissus cascavella*, venom [15] and sea anemone,* Aiptasia pallid*, nematocyst venom [29] also showed strict Ca^2+^ requirement [30].

PLA_2_ activity in the* Aedes albopictus *and* Culex quinquefasciatus *preparations revealed slightly higher enzyme activity in Tris buffer without additional calcium. This is in agreement with PLA_2_ from the midgut of tobacco hornworm [31] and* Aedes aegypti *larvae midgut [18].

Generally, enzyme activities are influenced by biophysical parameters, protein and substrate concentrations, pH, temperature, and reaction time. Increasing the amount of either enzyme or substrate generally will increase reaction rates because more active sites are available for reaction and more substrate molecules can bind with the active sites.

The* Aedes albopictus *and* Culex quinquefasciatus *preparations responded to the usual biophysical parameters in a way fairly similar to the* Aedes aegypti *preparations [18] except for its sensitivity to OOPC. PLA_2_ activity in* Aedes aegypti *[18] was less sensitive to OOPC than the* Aedes albopictus *and* Culex quinquefasciatus *preparations.* Aedes albopictus *PLA_2_ was inhibited at 5000 *μ*M of OOPC as compared to* Culex quinquefasciatus* PLA_2_, which was inhibited at a lower concentration of OOPC (500 *μ*M). In contrast, the PLA_2_ activity from* Aedes aegypti *[18] did not show any significant decrease in the presence of OOPC (5–5000 *μ*M).

Similar finding was also recorded for tobacco hornworm,* Manduca sexta *[31], digestive PLA_2_ where there was no inhibition in its enzyme activity when exposed to different concentrations of OOPC in a range of 5–500 *μ*M. On the other hand, the primary screwworm PLA_2_ preparation was more sensitive to OOPC, 50 *μ*M [25].

The* Aedes albopictus *and* Culex quinquefasciatus *PLA_2_ activity increased with time in reactions up to 1 hour, in agreement with PLA_2_ enzyme from other insects,* Aedes aegypti *[18] and* C. hominivorax *[25], and PLA_2_ from venoms,* Bothrops jararacussu *[22].

PLA_2_ from* Aedes albopictus* and* Culex quinquefasciatus *shares some similarity and differences with a related species,* Aedes aegypti*, where the optimal enzyme activity was at 40–50°C. However, the optimum pH condition differs.* Aedes aegypti* PLA_2_ activity was optimal at pH 9.0, which is similar with* Culex quinquefasciatus *PLA_2_, while for* Aedes albopictus *the enzyme activity declined at pH 9. Although the maximum enzyme activity differs, all mosquitoes PLA_2_ reaction studied slowed down in acidic conditions. This is in broad agreement with the pH conditions of insect midguts, which can be very high in lepidoptera and more acidic in mosquitoes [25].

This study has estimated the size of PLA_2_ from* Aedes albopictus *and* Culex quinquefasciatus* and provided information on partially purified PLA_2_ from crude homogenate of mosquito larval midgut by using Heparin column. In this study, both* Aedes albopictus* and* Culex quinquefasciatus *larval midgut PLA_2_ estimated molecular weights were found in a range of secretory insect PLA_2_. The molecular weights were estimated at 14.6–25 kDa and 25 kDa for* Aedes albopictus* and* Culex quinquefasciatus,* respectively. Distinct band at 25 kDa was shown in both PLA_2_ preparations of* Aedes albopictus *and* Culex quinquefasciatus* which are more likely the protein of interest in this study.

The estimated sizes agreed with the classic characteristic of sPLA_2_, which consists of small MW enzyme ranging from 13 to 15 kDa which were obtained from various organisms such as snake venoms, porcine pancreas, fungus, and bacteria [1] and for tiger beetle and human PLA_2_ which were reported to be 22 kDa and 55 kDa, respectively [19].

However, other investigations such as amino acid sequence and X-ray crystal structures of* Aedes albopictus* and* Culex quinquefasciatus* need to be conducted in order to classify the type of the digestive PLA_2_.

## 5. Conclusions

*Aedes albopictus* PLA_2_ from different sample preparations showed no significant difference in their activity, while for* Culex quinquefasciatus* significantly higher PLA_2_ in gut contents was shown if compared to other preparations. PLA_2_ from both enzymes did not require calcium (Ca^2+^) for full enzyme activity and both showed increasing of enzyme activity with increasing concentration of substrate. The PLA_2_ enzymatic assay from both mosquitoes showed accumulation of chromogenic substance up to 60 minutes of incubation time at 40°C. Both enzymes reacted in full catalytic activity in alkaline condition.* Aedes albopictus* PLA_2_ was significantly inhibited by site specific PLA_2_ inhibitor, OOPC. However,* Culex quinquefasciatus *PLA_2_ was not significantly inhibited by the same inhibitor. Based on the electrophoretic pattern of the enzyme samples, protein band at 20–25 kDa was observed in both mosquitoes. To conclude, there were no differences between the characteristic of PLA_2_ from* Aedes albopictus* and* Culex quinquefasciatus* except for its inhibition toward site specific inhibitor PLA_2_, OOPC, where the inhibitor does not affect the* Culex quinquefasciatus* PLA_2_ activity.

## Figures and Tables

**Table 1:** Mosquito larval preparation for localizing PLA_2_ enzyme experiment.

Groups	Larval sections
A	Gut-free bodies (40 individual larval bodies/pool)
B	Gut and content (40 individual guts/pool)
C	Isolated gut contents (40 individual isolated gut contents/pool)

## Data Availability

The data for the findings of this study will be available upon request to any of this article's authors.

## References

[1] Burke J. E., Dennis E. A. (2009). Phospholipase A_2_ biochemistry.

[2] Birts C. N., Barton C. H., Wilton D. C. (2010). Catalytic and non-catalytic functions of human IIA phospholipase A_2_.

[3] Hoxha E., Harendza S., Zahner G. (2011). An immunofluorescence test for phospholipase-A_2_-receptor antibodies and its clinical usefulness in patients with membranous glomerulonephritis.

[4] Aboshi T., Shimizu N., Nakajima Y. (2013). Biosynthesis of linoleic acid in Tyrophagus mites (Acarina: Acaridae).

[5] Blaul B., Steinbauer R., Merkl P., Merkl R., Tschochner H., Ruther J. (2014). Oleic acid is a precursor of linoleic acid and the male sex pheromone in *Nasonia vitripennis*.

[6] Brandstetter B., Ruther J. (2016). An insect with a delta-12 desaturase, the jewel wasp nasonia vitripennis, benefits from nutritional supply with linoleic acid.

[7] Stanley‐Samuelson D. W., Jurenka R. A., Cripps C., Blomquist G. J., de Renobales M. (1988). Fatty acids in insects: Composition, metabolism, and biological significance.

[8] Jurenka R. A., Stanley-Samuelson D. W., Loher W., Blomquist G. J. (1988). De novo biosynthesis of arachidonic acid and 5,11,14-eicosatrienoic acid in the cricket Teleogryllus commodus.

[9] Stanley D., Kim Y. (2014). Eicosanoid Signaling in Insects: From Discovery to Plant Protection.

[10] Six D. A., Dennis E. A. (2000). The expanding superfamily of phospholipase A_2_ enzymes: classification and characterization.

[11] Jiménez M., Cabanes J., Gandi F. (2003). A continuous spectrophotometric assay for phospholipase A_2_ activity.

[12] Tang C. S., Lam-Phua S. G., Chung Y. K., Giger A. D. (2007). Evaluation of a grass infusion-baited autocidal ovitrap for the monitoring of Aedes aegypti (L.).

[13] Ghani A. A.

[14] Nenad P., Carolyn G., Paul E. L., Neil L. A., Misso L. A., Thompson P. J. (2001). A simple assay for a human serum phospholipase A2 that is associated with high-density lipoproteins.

[15] Beghini D. G., Toyama M. H., Hyslop S., Sodek L., Novello J. C., Marangoni S. (2000). Enzymatic characterization of a novel phospholipase A_2_ from Crotalus durissus cascavella rattlesnake (Maracambòia) venom.

[16] Schägger H., von Jagow G. (1987). Tricine-sodium dodecyl sulfate-polyacrylamide gel electrophoresis for the separation of proteins in the range from 1 to 100 kDa.

[17] Gromova I., Celis J. E. (2006). Protein detection in gels by silver staining: a procedure compatible with mass-spectrometry.

[18] Nor Aliza A. R., Stanley D. W. (1998). A digestive phospholipase A_2_ in larval mosquitoes, Aedes aegypti.

[19] Schaloske R. H., Dennis E. A. (2006). The phospholipase A_2_ superfamily and its group numbering system.

[20] Bell J. D., Sanchez S. A., Hazlett theordore L. (2003). Liposomes in the study of PLA2 activity.

[21] Stanley D. (2006). The non-venom insect phospholipases A_2_.

[22] Bonfim V. L., Toyama M. H., Novello J. C. (2001). Isolation and enzymatic characterization of a basic phospholipase A_2_ from *Bothrops jararacussu* snake venom.

[23] Martins W., Baldasso P. A., Honório K. M. (2014). A novel phospholipase A_2_ (D49) from the venom of the Crotalus oreganus abyssus (North American Grand Canyon rattlesnake).

[24] Maruñak S. L., Leiva L., Garcia Denegri M. E., Teibler P., Acosta De Pérez O. (2008). Isolation and biological characterization of a basic phospholipase A_2_ from Bothrops jararacussu snake venom.

[25] Nor Aliza A. R., Rana R. L., Skoda S. R., Berkebile D. R., Stanley D. W. (1999). Tissue polyunsaturated fatty acids and a digestive phospholipase A2 in the primary screwworm, Cochliomyia hominivorax.

[26] Fleer E. A. M., Puijk W. C., Slotboom A. J., de Haas G. H. (1981). Modification of Arginine Residues in Porcine Pancreatic Phospholipase A2.

[27] Uscian J. M., Miller J. S., Howard R. W., Stanley-Samuelson D. W. (1992). Arachidonic and eicosapentaenoic acids in tissue lipids of two species of predacious insects, Cicindela circumpicta and Asilis sp..

[28] Uscian J. M., Miller J. S., Sarath G., Stanley-Samuelson D. W. (1995). A digestive phospholipase A_2_ in the tiger beetle Cicindella circumpicta.

[29] Grotendorst G. R., Hessinger D. A. (2000). Enzymatic characterization of the major phospholipase A_2_ component of sea anemone (Aiptasia pallida) nematocyst venom.

[30] Dennis E. A. (1994). Diversity of group types, regulation, and function of phospholipase A_2_.

[31] Rana R. L., Sarath G., Stanley D. W. (1998). A digestive phospholipase A_2_ in midguts of tobacco hornworms, Manduca sexta L.

